# Evaluation of Arteriolar Smooth Muscle Cell Function in an *Ex Vivo* Microvascular Network Model

**DOI:** 10.1038/s41598-017-02272-4

**Published:** 2017-05-19

**Authors:** Jessica M. Motherwell, Mohammad S. Azimi, Kristine Spicer, Natascha G. Alves, Nicholas A. Hodges, Jerome W. Breslin, Prasad V. G. Katakam, Walter L. Murfee

**Affiliations:** 1 Tulane University, Department of Biomedical Engineering, New Orleans, LA 70118 United States; 2 University of South Florida, Department of Molecular Pharmacology and Physiology, Tampa, FL 33612 United States; 3 Tulane University, Department of Pharmacology, New Orleans, LA 70112 United States

## Abstract

An emerging challenge in tissue engineering biomimetic models is recapitulating the physiological complexity associated with real tissues. Recently, our laboratory introduced the rat mesentery culture model as an *ex vivo* experimental platform for investigating the multi-cellular dynamics involved in angiogenesis within an intact microvascular network using time-lapse imaging. A critical question remains whether the vessels maintain their functionality. The objective of this study was to determine whether vascular smooth muscle cells in cultured microvascular networks maintain the ability to constrict. Adult rat mesenteric tissues were harvested and cultured for three days in either MEM or MEM plus 10% serum. On Day 0 and Day 3 live microvascular networks were visualized with FITC conjugated BSI-lectin labeling and arteriole diameters were compared before and five minutes after topical exposure to vasoconstrictors (50 mM KCl and 20 nM Endothelin-1). Arterioles displayed a vasoconstriction response to KCl and endothelin for each experimental group. However, the Day 3 serum cultured networks were angiogenic, characterized by increased vessel density, and displayed a decreased vasoconstriction response compared to Day 0 networks. The results support the physiological relevance of the rat mesentery culture model as a biomimetic tool for investigating microvascular growth and function *ex vivo*.

## Introduction

The tissue engineering of biomimetic models offers valuable opportunities for pre-clinical therapy evaluation and fundamental physiology research. A challenge, however, is mimicking or building the multi-scale complexity of a real tissue. Consider microvascular remodeling, which is a complex biological process that involves multiple cell types, intracellular signaling, cell-cell interactions, extracellular matrix proteins, and soluble factors^1^. Microvascular remodeling, including angiogenesis or the growth of new blood vessels, is involved in numerous pathological conditions (e.g. tumor growth and metastasis, myocardial infarction, ischemia, and wound healing)^2, 3^. This has motivated the development of two-dimensional^4^, three-dimensional^5^, and microfluidic-based models^6^ to investigate the underlying cell, molecular, and environmental mechanisms associated with microvascular remodeling. Advances in these bottom-up approaches have made it possible to investigate cell-cell, cell-extracellular matrix, and hemodynamic stress interactions. However, there still exists a need to continue to incorporate the multi-cell, multi-system complexity associated with intact microvascular networks into current models to achieve greater physiological relevance.

Tissue culture approaches offer alternative top-down strategies to maintain the complexity of tissues *ex vivo* ^7–9^. As an example of a top-down tissue culture strategy, our laboratory recently introduced the rat mesentery culture model to investigate cell-cell interactions during microvascular remodeling in viable, intact microvascular networks^10^. We have previously demonstrated that this model can be used to investigate angiogenesis^10^, lymphangiogenesis^11^, pericyte-endothelial cell interactions during capillary sprouting^10^, and anti-angiogenic drug effects^12^. The time-lapse capability as well as the innate complexity of the mesentery tissue, which includes blood vessels, lymphatic vessels, and interstitial cells, highlight the advantages of the rat mesentery culture model. However, a critical question is to what extent the smooth muscle cells remain functional.

The objective of this study was to determine if the smooth muscle cells (SMCs) within the rat mesenteric microvascular network maintain the ability to constrict during culture. Herein we examined receptor mediated and non-receptor mediated vasoconstriction responses by arterioles. Our results suggest that 1) SMCs can maintain the ability to constrict after three days in culture, and 2) media supplementation with serum cause angiogenesis and a decrease in contractile function. Our *ex vivo* characterization of cultured tissues is validated by intravital microscopy comparison of microvascular networks from unstimulated adult rats and rats that were stimulated to undergo angiogenesis. The findings from this study provide new information regarding the physiological relevance of the rat mesentery culture model as a tool to investigate microvascular growth and function within an intact microvascular network and support the potential of top-down tissue culture methods for biomimetic tissue engineering applications. The identification of impaired vasoconstriction in networks characterized by angiogenesis based on our culture model data and chronic *in vivo* data further support a novel discovery for angiogenesis research. The observation of impaired function in remodeling vessels is new or at least underappreciated and motivates a platform for future investigation.

## Results

### Vascular smooth muscle cells maintain the ability to constrict in cultured *ex vivo* rat mesenteric tissues

**Figure 1:**
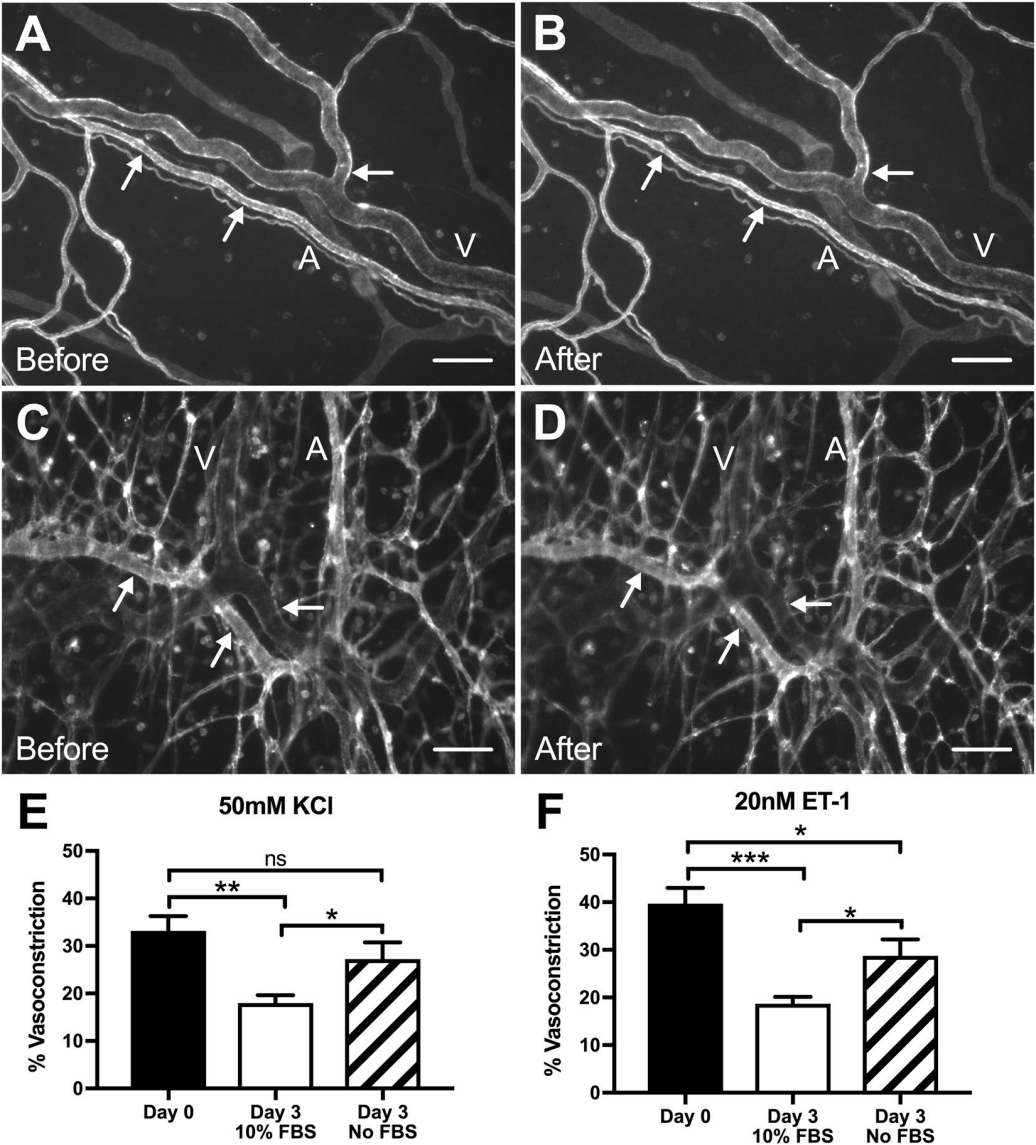
Comparison of arteriole vasoconstriction responses to 50 mM KCl and 20 nM ET-1 between Day 0 (pre-culture) and Day 3 (cultured) *ex vivo* tissues. (**A–D**) Comparison between Day 0 (**A**,**B**) and Day 3 + 10% FBS (**C**,**D**) groups before and after drug exposure demonstrates arteriole constriction in rat mesenteric tissues. Microvascular networks were visualized with BSI-lectin labeling. Arrows indicate points of constriction along the vessel. Scale bars = 100 μm. A = arteriole, V = venule. (**E**,**F**) Percent vasoconstriction was quantified after five-minute exposure to 50 mM KCl and 20 nM ET-1, respectively. Black, white, and striped bars represent Day 0, Day 3 + 10% FBS, and Day 3 + No FBS groups respectively. *, **, and *** indicates a significant difference of p < 0.05, p < 0.01, and p < 0.001 respectively by One-Way ANOVA and Student-Newman-Keuls post hoc method. “ns” Indicates no significant difference (p > 0.05).

**Figure 2:**
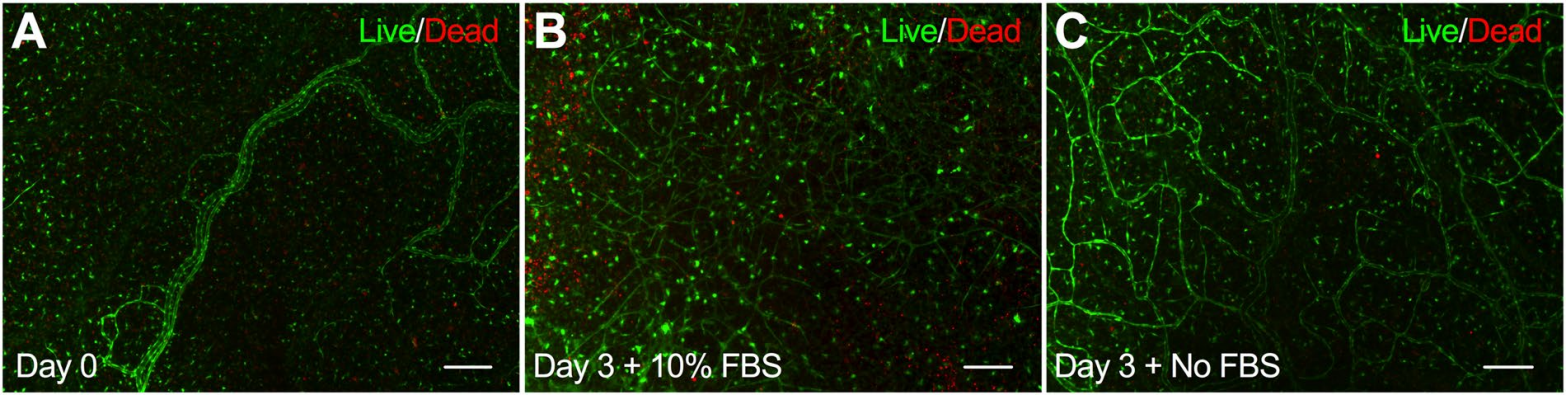
Qualitative evaluation of *ex vivo* tissue viability between Day 0 (pre-culture) and Day 3 (cultured) mesenteric tissues. (**A–C**) Cell viability/cytotoxicity labeling with Calcein AM (green) and Ethd-1 (red) confirms tissues remain viable up to three days in the rat mesentery culture model. Scale bars = 200 μm. A = arteriole, V = venule.

BSI-lectin labeling of Day 0 (pre-culture) and Day 3 (culture) groups identified endothelial cells (ECs) along the blood and lymphatic networks within the rat mesenteric microvasculature (Fig. 1A–D). Arteriole constriction in Day 0 mesenteric tissues was observed for both 50 mM KCl (33 ± 3.1%; n = 10) and 20 nM endothelin-1 (ET-1) (40 ± 3.3%; n = 17) vasoconstrictors (Fig. 1E and F). Comparison of arteriole constriction between Day 0 and Day 3 + 10% FBS groups revealed a significant decrease in contractile response to both 50 mM KCl (18 ± 1.6%; n = 13, p = 0.002) and 20 nM ET-1 (19 ± 1.4%; n = 16, p < 0.001). Arterioles from Day 3 + No FBS group displayed a modest but significant decrease in contractile response to 20 nM ET-1 (29 ± 3.5%; n = 14, p = 0.012) compared to Day 0 responses, however there was no significant difference in contractile response to 50 mM KCl (27 ± 3.5%; n = 12, ns). Justification for the KCl and ET-1 concentrations is supported by the literature^13–15^ and representative concentration-dependent response curves from arterioles in Day 0 mesenteric tissues (see Supplementary Fig. S1). Additional sham control experiments for Day 0, Day 3 + 10% FBS, and Day 3 + No FBS groups were performed in the absence of KCl and ET-1 vasoconstrictors. The negligible response to the sham controls confirmed that the arteriole constriction responses were due to the presence of vasoconstrictors (see Supplementary Fig. S2). Evaluation of viability/cytotoxicity labeling confirmed pre-culture (Day 0) and cultured (Day 3 + 10% FBS and Day 3 + No FBS) mesenteric tissues remained alive (Fig. 2A–C).

**Figure 3:**
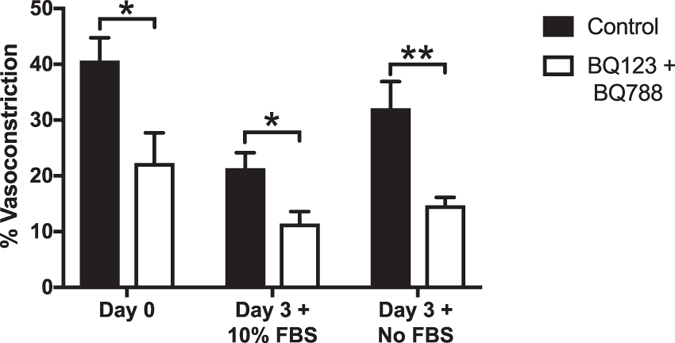
Evaluation of endothelin-1 sub-type receptors (ET_A_ and ET_B_) in the rat mesentery culture model. Comparison of arteriole constriction responses to 20 nM ET-1 in the presence of BQ-123 and BQ-788 antagonists. The statistically significant difference between Control and BQ-123 + BQ-788 for Day 0 (pre-culture) and Day 3 (cultured) groups suggests the endothelin-1 sub-type receptors (ET_A_ and ET_B_) remain functional in the rat mesentery culture model after three days in culture. Black and white bars represent the Control and BQ-123 + BQ-788 groups respectively. * and ** indicates a significant difference of p < 0.05 and p < 0.01 by two-tailed Student’s t-test.

ET_A_ and ET_B_ receptor sub-types are present on SMCs and both mediate ET-1 induced vasoconstriction^15–17^. For this study, BQ-123 (ET_A_) and BQ-788 (ET_B_) antagonists were combined in order to inhibit ET-1 vasoconstriction via SMCs and vascular ECs. In order to further confirm cellular mechanisms of action, the effects of BQ-123 and BQ-788 ET-1 receptor antagonists on ET-1 induced contractility in Day 0, Day 3 + 10% FBS, and Day 3 + No FBS groups were analyzed. The combined presence of BQ-123 and BQ-788 resulted in significantly reduced arteriole constriction in all three groups (Day 0: 23 ± 5.4%; p = 0.019, Day 3 + 10% FBS: 11 ± 4.0%; p = 0.013, Day 3 + No FBS: 15 ± 1.4%; p = 0.003) compared to 20 nM ET-1 control groups (Day 0: 41 ± 4.1%, Day 3 + 10% FBS: 21 ± 2.9%, Day 3 + No FBS: 32 ± 4.9%;) (Fig. 3).

**Figure 4:**
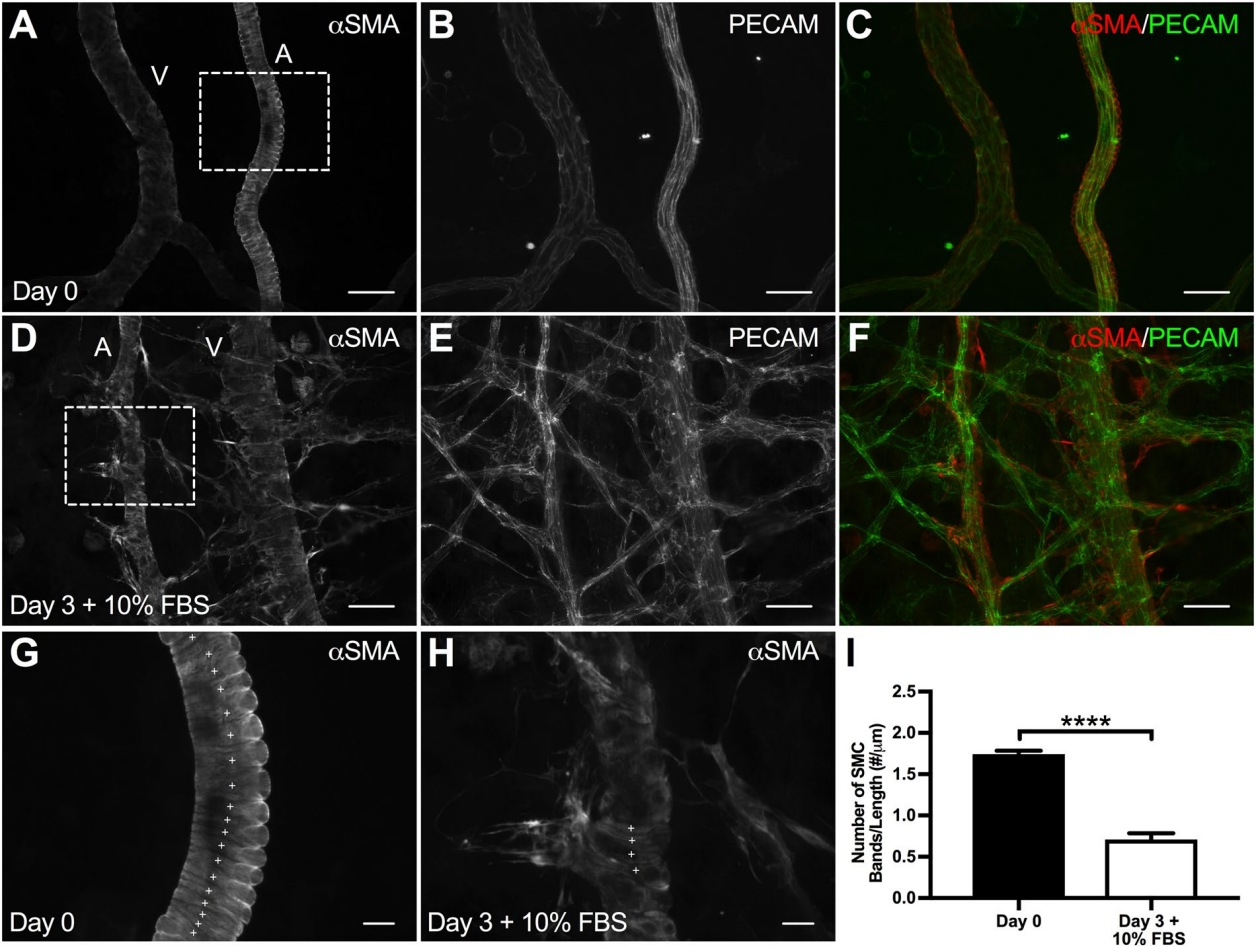
Evaluation of smooth muscle cell morphology in the rat mesentery culture model. (**A–F**) Comparison of Day 0 and Day 3 + 10% FBS SMC morphology. PECAM and αSMA labeling identified increased EC sprouting and decreased SMC bands after three days in culture with 10% FBS compared to bands in Day 0 tissues. Scale bars = 50 μm. A = arteriole, V = venule. (**G–I**) Evaluation of SMC bands, where (**G**,**H**) are higher magnifications of the tissue regions indicated by the squares shown in (**A**) and (**D**) respectiveley. Comparison of Day 0 and Day 3 + 10% FBS tissues reveals a decrease in SMC bands after three days in culture with serum. Plus signs identify SMC bands. Scale bars = 10 μm. Black and white bars represent Day 0 and Day 3 + 10% FBS groups respectively. **** indicates a significant difference of p < 0.0001 by two-tailed Student’s t-test.

**Figure 5:**
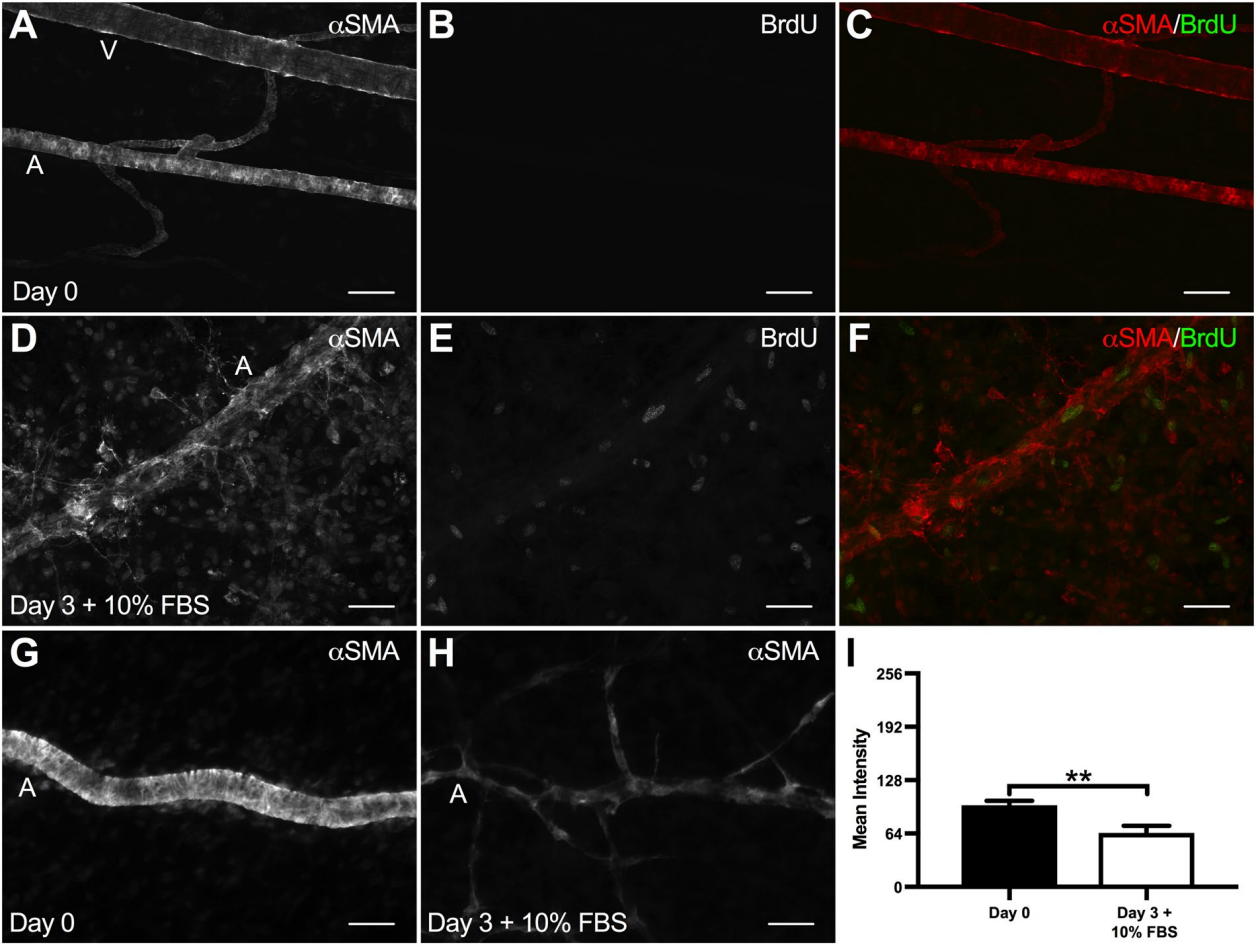
Evaluation of smooth muscle cell phenotype in the rat mesentery culture model. (**A–F**) Comparison of Day 0 and Day 3 + 10% FBS SMC proliferation. BrdU and αSMA labeling identified increased vascular cell proliferation after three days in culture with 10% FBS compared to Day 0 tissues. Scale bars = 50 μm. A = arteriole, V = venule. (**G–I**) Evaluation of αSMA labeling intensity. (**G**,**H**) Representative αSMA labeling for Day 0 and Day 3 + 10% FBS tissues. Scale bars = 50 μm. A = arteriole. (**I**) Comparison of Day 0 and Day 3 + 10% FBS tissues reveals a decrease in αSMA labeling intensity after three days in culture with serum. Black and white bars represent Day 0 and Day 3 + 10% FBS groups respectively. ** indicates a significant difference of p < 0.01 by two-tailed Student’s t-test.

A potential mechanism of the impaired vasoconstriction response along arterioles is SMC morphology and/or phenotypic alterations^1^. After three days in culture, arteriolar SMCs in the angiogenic networks (Fig. 4D–F) qualitatively displayed decreased αSMA-positive cell wrapping along arterioles and venules compared to pre-culture (Day 0) tissues (Fig. 4A–C). Quantification of αSMA-positive SMC bands between Day 0 and Day 3 + 10% FBS tissues revealed a significant decrease (p < 0.0001) in SMC wrapping after three days in culture with serum (Fig. 4G–I). In order to evaluate SMC proliferation along arterioles, additional tissues were labeled with BrdU and αSMA. Qualitative evaluation of BrdU-positive labeling revealed an increase in vascular cell proliferation in the Day 3 + 10% FBS group compared to the Day 0 group (Fig. 5A–F). Quantitative analysis of αSMA labeling mean intensity revealed a significant decrease (p = 0.0035) between Day 0 and Day 3 + 10% FBS groups (Fig. 5G–I).

**Figure 6:**
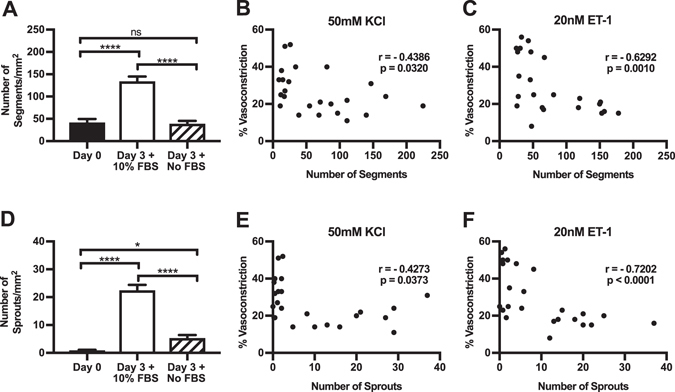
Assessment of the relationship between angiogenesis and vasoconstriction responses. (**A–F**) Evaluation of vascular vessel segments (**A–C**) and vessel sprouts (**D–F**). Correlation of segments (**B**,**C**) and sprouts (**E**,**F**) from vasoconstriction responses to 50 mM KCl and 20 nM ET-1. * and **** indicates a significant difference of p < 0.05 and p < 0.001 respectively, by One-Way ANOVA and Student-Newman-Keuls post hoc method. “ns” Indicates no significant difference (p > 0.05). “r” Indicates the Spearman rank order correlation coefficient. Black, white, and striped bars represent Day 0, Day 3 + 10% FBS, and Day 3 + No FBS groups respectively.

The changes in smooth muscle contractility were also correlated with angiogenesis. Comparisons of vessel segments and sprouts indicate a significant increase in vessel density and capillary sprouting from the Day 3 + 10% FBS group (134 ± 10.4 segments/mm^2^, n = 16; and 23 ±1.9 sprouts/mm^2^, n = 16) in comparison to Day 0 (43 ± 7.4 segments/mm^2^, n = 16, p < 0.0001; and 1.0 ± 0.2 sprouts/mm^2^, n = 16, p < 0.0001) and Day 3 + No FBS (39 ± 6.2 segments/mm^2^, n = 16, p < 0.0001; and 5.3 ± 1.1 sprouts/mm^2^, n = 16, p < 0.0001) groups (Fig. 6A and D). Vessel sprouts from the Day 3 + No FBS group were significantly different (p = 0.020) compared to the Day 0 group. However, vessel segments were not significantly different. A spearman’s correlation was run to determine the relationship between vascular segments, vascular sprouts, and vasoconstriction responses to 50 mM KCl and 20 nM ET-1, respectively (Fig. 6B and C,E,F). There was a strong, negative monotonic correlation between 20 nM ET-1 vasoconstriction responses and vascular segments (r = −0.6292, n = 24, p = 0.0010) as well as vascular sprouts (r = −0.7202, n = 24, p < 0.0001) (Fig. 6C and F). Additionally, there was a negative monotonic correlation between 50 mM KCl vasoconstriction responses and vascular segments (r = −0.4386, n = 24, p = 0.0320) and vascular sprouts (r = −0.4273, n = 24, p = 0.0373) (Fig. 6B and E).

### Vascular smooth muscle cell contractile function decreases during angiogenesis within *in vivo* rat mesenteric tissues

**Figure 7:**
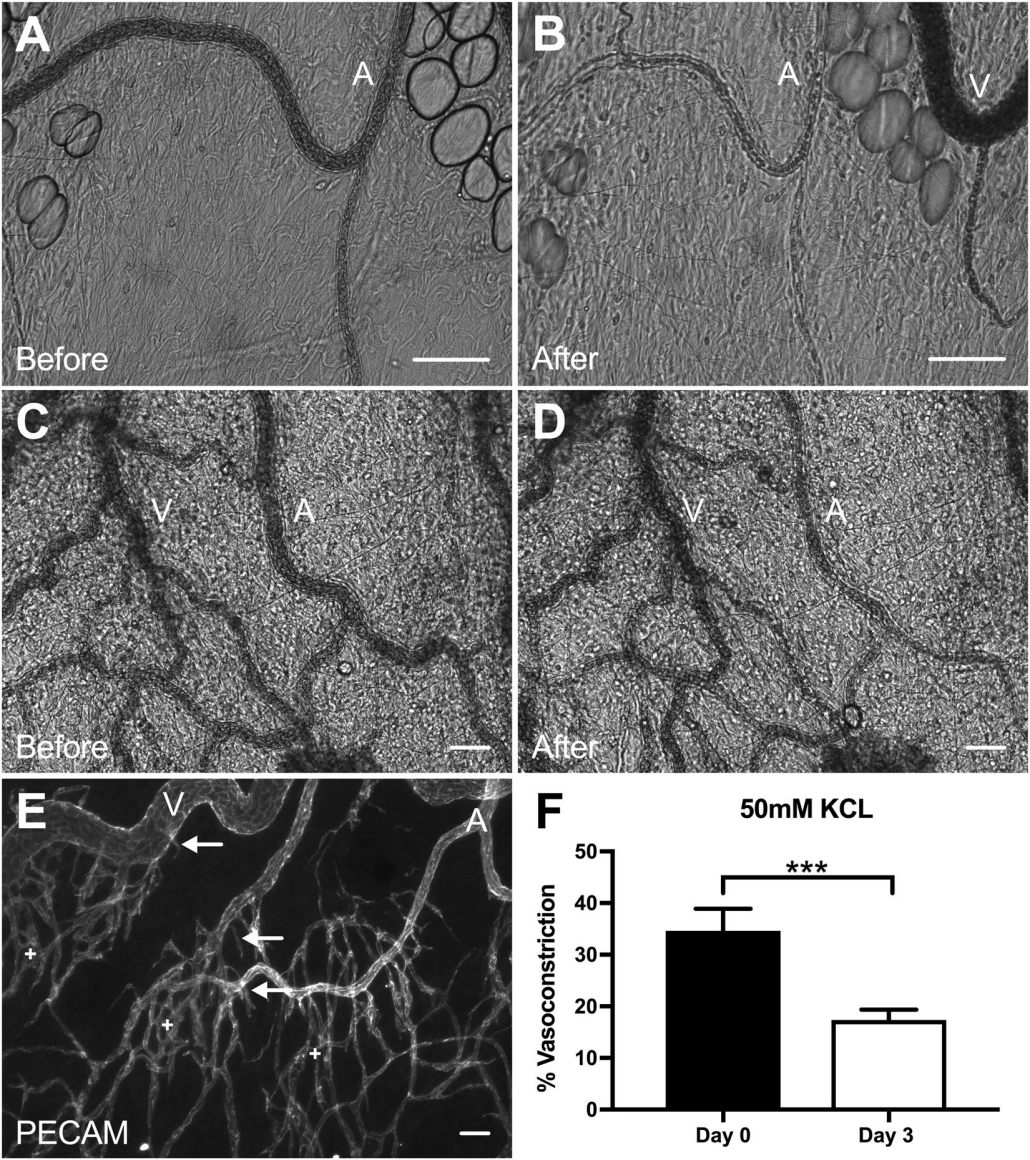
Evaluation of intravital arteriole vasoconstriction response to 50 mM KCl. (**A–D**) Comparison of Day 0 (**A**,**B**) and angiogenic Day 3 (**C**,**D**) mesenteric tissue responses to 50 mM KCl. Scale bars = 50 μm. A = arteriole, V = venule. (**E**) PECAM labeling identified EC sprouts along blood vessels confirming Day 3 tissues were undergoing angiogenesis. Arrows represent EC sprouts and plus signs represent capillaries. Scale bars = 50 μm. A = arteriole, V = venule. (**F**) Day 3 angiogenic arterioles exhibited a statistically significant difference (p < 0.001) in vasoconstriction compared to Day 0 responses. Black and white bars represent Day 0 and angiogenic Day 3 groups respectively. *** indicates a significant difference of p < 0.001 by two-tailed Student’s t-test.

Maintained vessel function along arterioles in the *ex vivo* cultured tissues is supported by the observation of similar responses to KCl during intravital microscopy. No significant difference was observed between the freshly harvested Day 0 (pre-culture) tissues (33 ± 3.1%; n = 10) (Fig. 1A and B) and the unstimulated Day 0 adult tissues constricted intravitally (35 ± 4.3%; n = 13) (Fig. 7A and B). Interestingly, arterioles in the mesentery exteriorization stimulated networks displayed a significant decrease (p = 0.003) in contractility between Day 0 (35 ± 4.3%; n = 13) and angiogenic Day 3 (stimulated) (17 ± 2.0%; n = 21) tissues (Fig. 7F) This phenomenon was consistent with the observations made in our tissue culture experiments. Angiogenesis in the *in vivo* stimulated tissues was confirmed by the dramatic increase in PECAM-positive vessel density, capillary sprouting, and vessel tortuosity consistent with previous characterizations of the mesentery exteriorization model^10, 11^. Additionally, PECAM labeling of Day 3 (stimulated) tissues post-KCl stimulation identified ECs along arterioles, venules, and capillaries and confirmed vascular networks were undergoing angiogenesis (Fig. 7E).

## Discussion

The main contribution of this study is the establishment of the rat mesentery culture model as an *ex vivo* experimental platform in which SMCs remain functional. To our knowledge, this is the first demonstration of a culture model that contains functional SMCs along arterioles within intact microvascular networks. Our initial developments of the rat mesentery culture model demonstrated the ability to stimulate angiogenesis, lymphangiogenesis, and to investigate pericyte-endothelial cell interactions^10, 11^. More recently, we have established the ability to incorporate time-lapse imaging into the rat mesentery culture model as a tool to evaluate anti-angiogenic drug effects at multiple time-points in real time^12^. This study expands upon our previous work by utilizing time-lapse imaging to confirm that the contractile function of SMCs is maintained along arterioles.

Vascular responses in mesenteric tissues were examined by testing the effects of two vasoconstrictors, KCl and ET-1. KCl and ET-1 were selected as vasoconstrictors for this study because their effects on rat mesenteric vessels is well-documented in the literature^14, 18–20^. KCl is used for testing the viability of excised vessels for physiological vasoreactivity studies^13, 14^ and ET-1 is a potent vasoconstrictor derived from ECs^21^. Notably, KCl and ET-1 elicit vasoconstriction by diverse mechanisms. KCl promotes vasoconstriction by non-receptor mediated mechanisms and ET-1 induces vasoconstriction by activating specific endothelin receptor-linked signaling pathways. Thus, the two pharmacological agents afford comprehensive examination of the contractile properties of SMCs in the rat mesentery culture model. In this study, we observed that topical exposure to both 50 mM KCl and 20 nM ET-1 produced constriction responses in pre-culture (Day 0) and cultured (Day 3) arterioles. Importantly, sham experiments per group (Day 0, Day 3 + 10% FBS, and Day 3 + No FBS) confirmed that the normal vasoconstriction behavior in the absence of KCl and ET-1 was negligible (see Supplementary Fig. S2). The vasoconstrictor concentrations were chosen based on established values from the literature^13–15^ and evaluation of the concentration-response curves (see Supplementary Fig. S1). We also observed arteriole constriction in response to other vasoconstrictors including norepinephrine and phenylephrine (data not shown).

**Figure 8:**
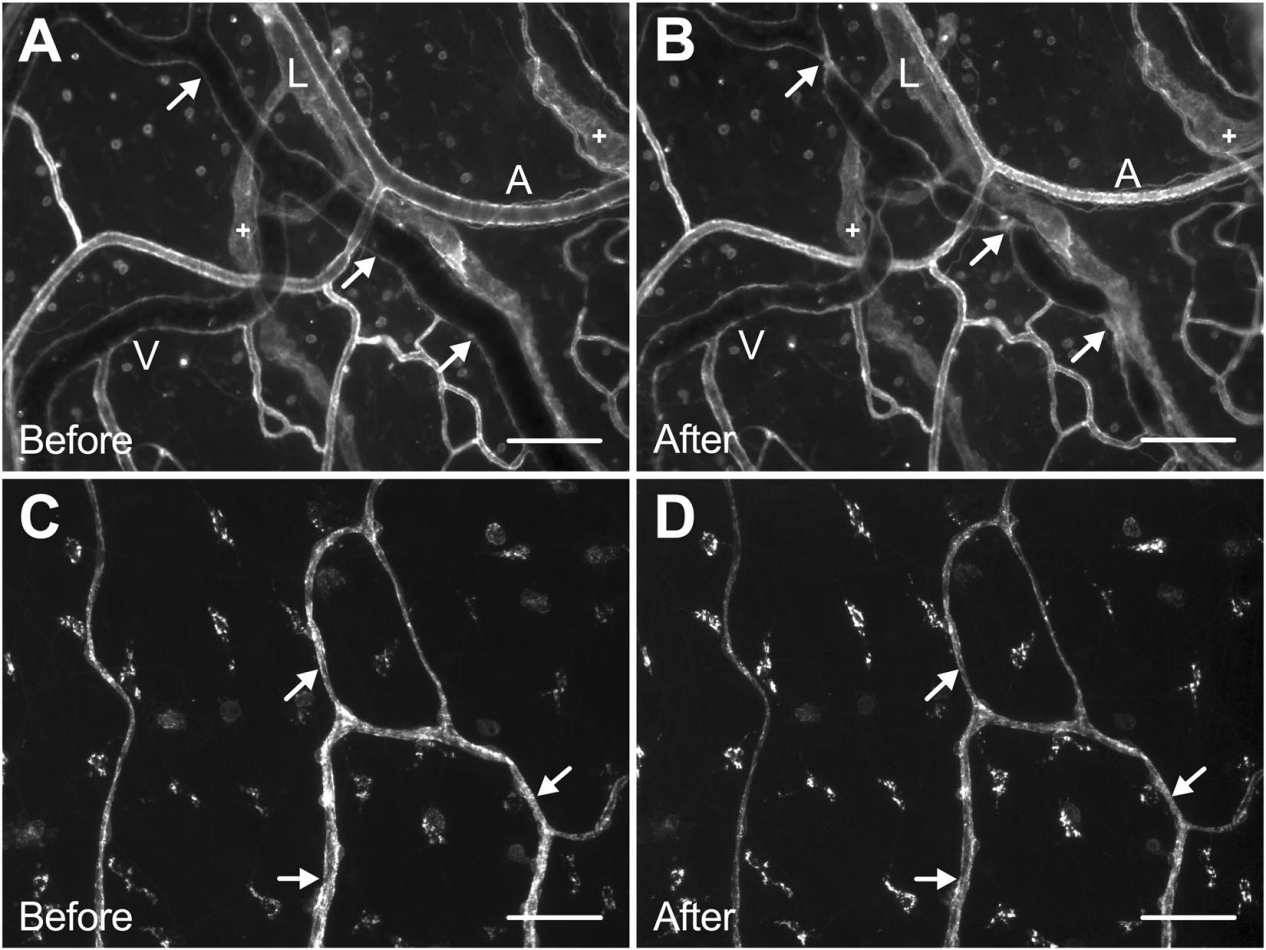
Qualitative evaluation of vasoconstriction responses from venules, initial lymphatics, and capillaries in Day 0 *ex vivo* tissues. (**A**,**B**) Comparison of before and after images demonstrates venule vasoconstriction response to 20 nM ET-1 and a lack of constriction from initial lymphatics, indicated by the plus signs. Similar responses were observed with 50 mM KCl exposure (data not shown). Microvascular networks were visualized with BSI-lectin labeling. Arrows indicate points of constriction. Scale bars = 100 μm. A = arteriole, V = venule, L = lymphatic. (**C**,**D**) Capillary vasoconstriction responses to 50 mM KCl was observed. Microvascular networks were visualized with BSI-lectin labeling. Arrows indicate points of constriction. Scale bars = 50 μm.

An advantage of the model is its simplicity. The tissue is self-contained, easy to obtain, and does not require embedding in a matrix. Another advantage is that in cultured rat mesenteric tissues, the microvascular networks remain intact^10^. This characteristic motivates future studies to evaluate how vasoreactive responses might vary along a network. Observation of vasoconstriction responses along venules and capillaries (Fig. 8) and the observed lack of contraction in response to KCl and ET-1 along initial lymphatics (Fig. 8A and B), characterized by a lack of SMC wrapping, supports the potential power of the model for investigating the mechanisms associated with vessel-type specific responses. At present, however, the probing of mechanisms is limited to pharmacological, immunolabelling or siRNA approaches as a limitation of our model is the lack of available transgenic rats in comparison to mouse models. Unfortunately the mouse mesentery is avascular^22^, which eliminates its use as a substitution.

For our analysis of *ex vivo* tissues, the percentage of vasoconstriction was measured at the point of maximum constriction along the arteriole. While constriction was consistent along the length of arterioles, the magnitude of constriction was sometimes heterogeneous. We speculate that localized pinching could be due to local differences in SMC structure, yet the potential spatially specific responses along a single vessel or across vessel types, for that matter, warrant further investigation.

Our observation of impaired arteriolar constriction in the Day 3 + 10% FBS group suggests that the culture conditions and/or angiogenesis have an effect on SMC functionality. Given that we have previously demonstrated that supplementing media with 10% FBS creates a robust angiogenic response in the rat mesentery after three days in culture^12^, we hypothesized that the decreased vessel constriction was influenced by the angiogenic state of the tissue. To test this hypothesis, tissues were cultured for three days without serum and topically administered KCl and ET-1. Interestingly, culture conditions alone without the stimulation of angiogenesis (i.e. serum supplementation) resulted in comparable vasoconstriction responses to Day 0 uncultured levels suggesting that mechanisms remain intact. These comparisons support that serum stimulation has an effect on SMC contractility (Fig. 1E and F) and indirectly supports the hypothesis that impaired constriction is associated with angiogenesis (Fig. 7F). More direct evidence for the alterations being associated with angiogenesis is provided by our intravital measurements for which vessel constriction in response to KCl was significantly decreased (p = 0.003) for angiogenic tissues. The identification of impaired vasoconstriction in networks characterized by angiogenesis based on our culture model data and chronic *in vivo* data support a novel finding for angiogenesis research. This discovery of impaired function in remodeling vessels is new and underappreciated in the fields of angiogenesis and tissue engineering. Moreover, it motivates a platform for future investigation of which mechanisms are altered in remodeling networks.

A potential cellular reason for the impaired vasoconstriction associated with angiogenic cultured networks might be SMC morphology or phenotype alterations. The observation of proliferating cells, decreased αSMA labeling intensity, and decreased SMC wrapping along Day 3 + 10% FBS arterioles implicates a transition to a more synthetic phenotype. In addition, SMCs along arterioles in microvascular networks cultured with serum can be characterized by class III-β tubulin upregulation, which we have previously reported as a marker of activated perivascular cells^23^. Additional studies are needed to investigate the mechanisms behind a possible phenotypic switch.

To further validate the functionality of SMCs in the rat mesentery culture model, a mechanism of action for the receptor-mediated vasoconstriction induced by ET-1 was confirmed using antagonists BQ-123 (ET_A_) and BQ-788 (ET_B_). The vasoactive effects of ET-1 are mediated through ET_A_ and ET_B_ sub-type receptors and their functions have been well-characterized. The ET_B_ receptor is found on both SMCs and vascular ECs. Smooth muscle ET_B_ receptor activation leads to vasoconstriction whereas endothelial ET_B_ receptor activation induces vasodilation via release of nitric oxide. In contrast, vascular smooth muscle ET_A_ receptor activation promotes vasoconstriction^21^. Since both ET_A_ and ET_B_ receptor binding on SMCs leads to constriction and our goal was to evaluate whether the constriction response in cultured arterioles was ET-1 receptor mediated, we blocked both receptors simultaneously via the combination of BQ-123 and BQ-788 receptor antagonists. While future studies will be needed to determine the individual effects of ET-1 sub-type receptor inhibition, our results suggest that SMCs in cultured tissues maintain the ET-1 signaling capability as a comparison of the ET-1 control group and BQ-123 + BQ-788 group revealed a significant decrease (p < 0.05) in arteriole constriction from all three experimental groups after exposure to 20 nM ET-1 for five minutes.

Evidence for maintained smooth muscle contractility in excised and subsequently cultured vessels is not new. For example, Bulley *et al*. have shown the ability to investigate vessel constriction in cerebral arteries after four days in culture^24^. Work by Clerin *et al*. have shown in vessel perfusion experiments that porcine arteries can maintain contractility out to twenty seven days in culture^25^ and Kapsokalyvas *et al*. have demonstrated rat mesenteric artery constriction after twenty four hours in culture using wire myography^26^. In comparison to these isolated vessel experiments, the current study demonstrates that arterioles within cultured microvascular networks can also maintain their ability to constrict. This result adds to our previous introduction of the rat mesentery culture model and establishes its novelty as an experimental platform to investigate the cellular dynamics during angiogenesis along the hierarchy of functional microvascular networks. We do not know of another model that matches this multi-scale complexity.

A current limitation of the rat mesentery culture model is the lack of flow in the microvascular network and the related effects of shear stress, which have been shown to regulate and guide angiogenesis *in vivo* ^27, 28^. The absence of flow in our model is a similar limitation to other common angiogenesis models including the aortic ring assay^9^, three-dimensional culture systems^5^, and two-dimensional cell cultures^4^. Recent developments in microfluidic devices have highlighted the importance of incorporating flow into *in vitro* models and future work will be needed to include flow into the rat mesentery culture model^29, 30^. In spite of the lack of flow, our confirmation of KCl and ET-1 induced vasoconstriction supports the maintained functionality of cultured SMCs along arterioles. Interestingly, observed examples of vasodilation in arterioles and venules from pre-cultured tissues following preconstriction with phenylephrine (data not shown), agrees with an acute evaluation of vasoreactivtiy in freshly harvested brain slices. Sagher *et al*. demonstrated that vasorelaxation of preconstricted vessels is possible, yet the dilated diameter did not increase past the resting state^13^. We speculate that the incorporation of flow and pressure is necessary to evaluate vasodilation responses past the resting vessel diameter. The incorporation of pressure would also enable the evaluation of the myogenic response and spontaneous tone, which for cannulated and pressurized arterioles *in vitro* can range from 30–40%^31^ compared to lower levels (less than 15%) associated with *in vivo* vessels from the same tissue-type^31^. A potential approach for incorporating flow and pressure would be vessel cannulation similar to the isolated vessel preparations. However, the number of network outputs and the levels of pressurization needed in upstream arterioles to maintain physiological flow levels throughout a network remain to be investigated.

In summary, our results suggest that SMCs within the cultured rat mesentery microvascular networks maintain their ability to constrict and that impaired vessel constriction is associated with angiogenesis. To our knowledge, this study is the first to demonstrate functional constriction of vessels within intact microvascular networks *ex vivo* and supports the novelty of the rat mesentery culture model as a biomimetic tool for microvascular research.

## Materials and Methods

### Rat mesentery tissue culture for time-lapse imaging

All animal experiments were approved by Tulane University’s Institutional Animal and Care Use Committee and performed in accordance with the US. Animal Welfare Act, US. Public Health Service Policy on the Humane Care and Use of Laboratory Animals, and the NIH *Guide for the Care and Use of Laboratory Animals*. Rat mesenteric tissues were harvested and cultured for time-lapse imaging according to our previous descriptions^10, 12^. Briefly, adult male Wistar rats (350–400 g) were anesthetized with an intramuscular injection of ketamine (80 mg/kg body weight) and xylazine (8 mg/kg body weight). An incision was made in the abdominal cavity and the mesentery was exteriorized using the ileum as a reference point. Mesentery tissues were spread onto a sterile plastic stage and the rat was euthanized with an intracardiac injection of 0.2 ml Beuthanasia. Vascularized mesenteric tissues were excised and rinsed once in sterile saline with 0.9% NaCl (Baxter; Deerfield, IL) and immersed in minimum essential media (MEM; Gibco; Grand Island, NY) containing 1% Penicillin-Streptomycin (PenStrep; Gibco; Grand Island, NY) at 37 °C. Tissues were then spread onto a thin polycarbonate filter fitted to a cell-crown insert (CellCrown; Sigma-Aldrich; St. Louis, MO). Each insert was inverted into individual wells of a 6-well culture plate and covered with 4 ml MEM with 1% PenStrep. Angiogenesis was stimulated by supplementing the media with 10% Fetal Bovine Serum (FBS; Gibco; Grand Island, NY). Tissues were randomized into the following groups: 1) Day 0, 2) Day 3 + 10% FBS, and 3) Day 3 + No FBS. Day 3 tissues were cultured in standard incubation conditions (5% CO_2_, 37 °C) for three days. Tissues for the Day 0 groups were stimulated the same day they were excised (t = 0).

### *Ex vivo* labeling for time-lapse imaging

All *ex vivo* studies followed the same labeling protocol to visualize the vasculature before conducting the experiment. Vascular networks were visualized by labeling with BSI-lectin conjugated to FITC (FITC-lectin; Sigma-Aldrich; St. Louis, MO). BSI-lectin labeling revealed blood and lymphatic vessel networks and vessel type was determined based on morphology. Each well was supplemented with FITC-lectin (1:40) and incubated for thirty minutes under standard conditions followed by two washes with lectin-free media. Arteriole (10–43 μm) and venule (12–50 μm) pairs feeding into the tissue from the border were identified within the vasculature for imaging.

### Image acquisition

Intravital images were acquired using a 10X (water, NA = 0.3) objective on an upright Nikon Eclipse LV100 microscope coupled with a Hamamatsu ORCA-Flash 2.8 camera. *Ex vivo* tissues and fixed immunohistochemicistry tissues were imaged using 4X (dry, NA = 0.1), 10X (dry, NA = 0.3), and 20X (oil, NA = 1.4) objectives on an inverted Olympus IX70 microscope coupled with a Photometrics CoolSNAP EZ camera.

### *Ex vivo* vasoconstriction study

All reagents were dissolved in a HEPES-buffered Physiological Saline Solution (HEPES-PSS) containing (in mM): 134 NaCl, 6 KCl, 1 MgSO_4_, 2 CaCl_2_, 10 HEPES, 0.026 EDTA, and 10 Glucose (pH 7.4).

For *ex vivo* vasoconstriction response studies, vessels were imaged on the day of exposure to vasoconstrictors according to the following experimental groups: 1) Day 0 (KCl; n = 10 tissues from 7 rats, ET-1; n = 17 tissues from 7 rats), 2) Day 3 + 10% FBS (KCl; n = 13 tissues from 8 rats, ET-1; n = 16 tissues from 10 rats), and 3) Day 3 + No FBS (KCl; n = 12 tissues from 2 rats, ET-1; n = 14 tissues from 2 rats). Tissues were topically exposed to either 50 mM KCl (Fisher Scientific; Fair Lawn, NJ) or 20 nM ET-1 (Sigma-Aldrich; St. Louis, MO) for five minutes, maintained at 37 °C and pH 7.4. Tissues were imaged throughout the duration with “Before” images taken before exposure and “After” images taken five minutes after exposure to vasoconstrictors.

### *Ex vivo* ET-1 antagonist study

Both the selective ET_A_ (BQ-123) and ET_B_ (BQ-788) receptor antagonists effect on ET-1 constriction were tested. Tissues were incubated with BQ-123 and BQ-788 the day of imaging according to the following experimental groups: 1) Day 0: n = 12 tissues from 2 rats, 2) Day 3 + 10% FBS: n = 8 tissues from 2 rats, and 3) Day 3 + No FBS: n = 8 tissues from 2 rats. Control groups were tested according to the following: 1) Day 0: n = 9 tissues from 2 rats, Day 3 + 10% FBS: n = 8 tissues from 2 rats, and Day 3 + No FBS: n = 8 tissues from 2 rats. BQ-123 and BQ-788 (Sigma-Aldrich; St. Louis, MO) were incubated with the tissues at a concentration of 3 μM each^17^ in HEPES-PSS, for thirty minutes prior to constriction. Control tissues were incubated in HEPES-PSS without the antagonists for thirty minutes prior to constriction. Tissues were topically administered 20 nM ET-1 for five minutes, maintained at 37 °C and pH 7.4. Vessels were imaged throughout the duration of exposure.

### Intravital vasoconstriction study

Intravital microscopy was performed on Day 0 tissues and on Day 3 tissues stimulated to undergo angiogenesis according to the following groups: 1) Day 0 (unstimulated): n = 13 tissues from 3 rats, and 2) Day 3 (stimulated): n = 21 tissues from 4 rats. For Day 0 tissues, adult male Wistar rats (350–400 g) were anesthetized with an intramuscular injection of ketamine (80 mg/kg body weight) and xylazine (8 mg/kg body weight). Using a sterile technique, an incision was made in the abdominal cavity and the mesentery was exteriorized onto a customized optical stage for microscopic observation. The exteriorized mesentery was superfused with HEPES-PSS at 37 °C and body temperature was maintained at 37 °C with a heating pad (Fine Science Tools; North Vancouver, BC). Vascular networks were visualized with brightfield microscopy and arterioles and venules were identified by observation of flow patterns at bifurcations. Tissues were topically administered 50 mM KCl for five minutes and vessels were imaged before and after exposure. To stimulate angiogenesis in the Day 3 (stimulated) tissues, rats were anesthetized as described above and the mesentery was exteriorized for 20 minutes. Exposed tissues were superfused with sterile saline at 37 °C and two centrally located vascularized tissues were marked with 7–0 silk sutures (Ethicon; Somerville, NJ). At the end of the 20-minute exteriorization, the mesentery was carefully returned to the abdominal cavity and the incision was sutured. The rats were allowed to recover from anesthesia and had access to food and water for the next three days. Buprenorphine was administered daily as a post-procedural analgesic. After three days, rats were anesthetized and the mesentery tissues were again exteriorized. The marked tissues were located and used as a reference point for the intravital vasoconstriction procedure described above for Day 0 (unstimulated) tissues. This exteriorization model is based on a previously published model of angiogenesis^32–34^ and was selected because it produces a robust multi-factorial remodeling response in mesenteric tissues. This response is characterized by a dramatic increase in capillary sprouting, vessel density and vessel tortuosity^32, 35^ over a short time course. While the exact mechanisms remain unclear, the exteriorization of the mesentery has been linked to mast cell activation and an increase of local histamine levels^36^.

### Quantification of vasoconstriction and angiogenesis

Vasoconstriction was defined as the point of maximum decrease in diameter along the length of a vessel within the field of view. Arterioles were identified based on EC morphology and location within the network^23^. Vessels were included in the quantification that were identified as arterioles with diameters larger than 10 μm. Additionally for intravital studies, arterioles versus venule identity was confirmed based on flow direction at vessel branch points. The following equation was used to calculate the percent of vasoconstriction = 100[(D_initial_ − D_final_)D_initial_^−1^], where D_initial_ is the vessel diameter before exposure to vasoconstrictors and D_final_ is the vessel diameter after exposure to vasoconstrictors. Image processing and quantification was done using the NIH Fiji open-source software version 2.0.0^37^. Before and after images were stacked and the straight line selection tool was used to measure the diameter of the vessel at the same location in the before (D_initial_) and after (D_final_) images. Diameter values were recorded using the measurement tool and exported to an excel spreadsheet for final quantification of percent vasoconstriction. Eight 4X images, each from the 50 mM KCl and 20 nM ET-1 groups, were randomly selected for analysis of the number of vessel segments and sprouts per area. Segments and sprouts were analyzed for the following groups: 1) Day 0: n = 16 tissues from 8 rats, 2) Day 3 + 10% FBS: n = 16 tissues from 9 rats, and 3) Day 3 + No FBS: n = 16 tissues from 4 rats. Vessel segments were defined as BSI-lectin-positive endothelial cells between two branch points and sprouts were defined as blind-ended segments originating from a vessel. Analysis was performed using the NIH Fiji open-source software version 2.0.0^37^, where segments and sprouts were counted using the Cell Counter plugin.

### Quantification of smooth muscle cell morphology

Blind analysis with triple replicates was performed to evaluate SMC bands per arteriole length. SMC bands were defined as αSMA-positive cells with bands wrapping around the circumference of the arteriole. Analysis was performed using the NIH Fiji open-source software version 2.0.0^37^, where SMC bands were counted using the Cell Counter plugin. The segmented line selection tool was used to calculate the length of the arteriole. SMC αSMA-positive bands were analyzed according to the following groups: 1) Day 0: n = 11 arterioles from 6 tissues from 4 rats, and 2) Day 3 + 10% FBS: n = 12 arterioles from 5 tissues from 3 rats.

### Quantification of αSMA mean intensity

Tissues were labeled for αSMA according to the follow groups: 1) Day 0: n = 10 tissues from 5 rats, and 2) Day + 10% FBS: n = 10 tissues from 4 rats. Tissues were imaged with the same exposure time (500 ms) using a 10X (dry, NA = 0.3) objective on an inverted Olympus IX70 microscope coupled with a Photometrics CoolSNAP EZ camera. Quantification was done using the NIH Fiji open-source software version 2.0.0, where the polygon selection tool was used to outline arterioles and the mean intensity of the selected area was measured.

### Immunohistochemistry

#### Viability/Cytotoxicity

Tissues were incubated with Calcein AM and EthD-1 (Invitrogen; Carlsbad, CA) in MEM for 10 minutes at 37 °C followed by two rinses with MEM.

#### αSMA/PECAM

Tissues were spread on microscope slides and fixed in methanol at −20 °C for thirty minutes and labeled according to the following protocol: 1) 1:200 mouse monoclonal biotinylated CD31 antibody (CD31 antibody, BD Pharmigen; San Diego, CA); 2) 1:500 CY2-conjugated streptavidin antibody (Jackson ImmunoResearch Laboratories; West Grove, PA) and 1:200 CY3-conjugated mouse monoclonal αSMA antibody (Sigma-Aldrich; St. Louis, MO).

#### αSMA/BrdU

Tissue culture media was replaced with MEM + 1% PenStrep supplemented with BrdU (1 mg/ml; Dako; Glostrup, Denmark) and incubated for two hours at 37 °C. After the incubation period, the BrdU solution was replaced with phosphate-buffered solution (PBS) with calcium and magnesium (Gibco; Grand Island, NY). Tissues were spread on microscope slides and fixed in methanol at −20 °C for thirty minutes and labeled according to the following protocol: 1) 6 N HCl for 1 hour at 37 °C; 2) 1:100 monoclonal mouse anti-bromodeoxyuridine (anti-BrdU) (Dako; Glostrup, Denmark) overnight at 4 °C; 3) 1:100 goat anti-mouse CY2 Fab fragments (Jackson Immunochemicals; West Grove, PA) for 1 hour at room temperature; 4) 1:200 CY3-conjugated mouse monoclonal αSMA antibody (Sigma-Aldrich; St. Louis, MO) for 1 hour at room temperature. All antibodies were diluted in antibody buffer solution (PBS + 0.1% saponin + 2% bovine serum albumin + 5% normal goat serum).

### Statistical analysis

Data are presented as mean ± standard error of mean (SEM). A two-tailed Student’s t-test was used to analyze the data. When comparing more than two means, one-way Analysis of Variance (ANOVA) followed by pairwise comparisons with the Student-Newman-Keuls method was used. Correlation analysis was performed using Spearman’s rank correlation. For all tests, a p-value < 0.05 was considered statistically significant. Statistical analysis was performed using SigmaStat version 3.5 and Prism version 7 software.

## Electronic supplementary material

Supplementary Information

Supplementary Video 2

Supplementary Video 1
