# Supplementary material for: Evaluation of Arteriolar Smooth Muscle Cell Function in an Ex Vivo Microvascular Network Model
Source: Sci Rep. 2017 May 19;7:2195. doi: 10.1038/s41598-017-02272-4 (PMC5438412; doi:10.1038/s41598-017-02272-4)
Supplement: Supplementary file 1 — Supplementary Information [file 41598_2017_2272_MOESM1_ESM.pdf]

Evaluation of Arteriolar Smooth Muscle Cell Function in an *Ex Vivo* Microvascular Network Model

Jessica M. Motherwell[1], Mohammad S. Azimi[1], Kristine Spicer[1], Natascha G. Alves[2], Nicholas A. Hodges[1], Jerome W. Breslin[2], Prasad V.G. Katakam[3], Walter L. Murfee[1]

[1]Tulane University, Department of Biomedical Engineering, New Orleans, LA, 70118, United States
[2]University of South Florida, Department of Molecular Pharmacology and Physiology, Tampa, FL, 33612, United States
[3]Tulane University, Department of Pharmacology, New Orleans, LA, 70112, United States

Materials and Methods

Concentration-response study

Six concentrations of ET-1 (10^{-12} – 10^{-6} M) and KCl (10^{-9} – 10^{-3} M) were tested on arterioles from Day 0 (pre-culture) tissues to construct a concentration-response curve. Vessels were topically exposed to increasing concentrations of ET-1 or KCl for five minute intervals with subsequent addition of drugs. Images were taken before and after exposure to each drug concentration. Concentration-response curves for KCl (n = 1 tissue from 1 rat) and ET-1 (n = 1 tissue from 1 rat) were constructed using sigmoidal curve fitting with Prism version 7 (GraphPad Software Inc., San Diego, CA, U.S.A.) to calculate the EC_{50} values (the effective concentration of KCl and ET-1 to elicit 50% of the maximum constriction response).

Ex vivo sham study

For *ex vivo* sham studies, vessels were imaged on the day of exposure to the sham according to the following experimental groups: 1) Day 0: n = 8 tissues from 2 rats, 2) Day 3 + 10% FBS: n = 8 tissues from 3 rats, and 3) Day 3 + No FBS: n = 8 tissues from 3 rats. For the sham study, culture media was replaced with HEPES-PSS without vasoconstrictors for five minutes, maintained at 37 °C and pH 7.4. Tissues were imaged throughout the five-minute duration of exposure to the sham. Afterwards, tissues were topically exposed to 50 mM KCl to ensure the vessel was responsive.

Statistical analysis

Data are presented as mean ± standard error of mean (SEM). A one-way Analysis of Variance (ANOVA) followed by pairwise comparisons with the Student-Newman-Keuls method was used to analyze the data. For all tests, a p-value < 0.05 was considered statistically significant. Statistical analysis was performed using SigmaStat version 3.5 software.

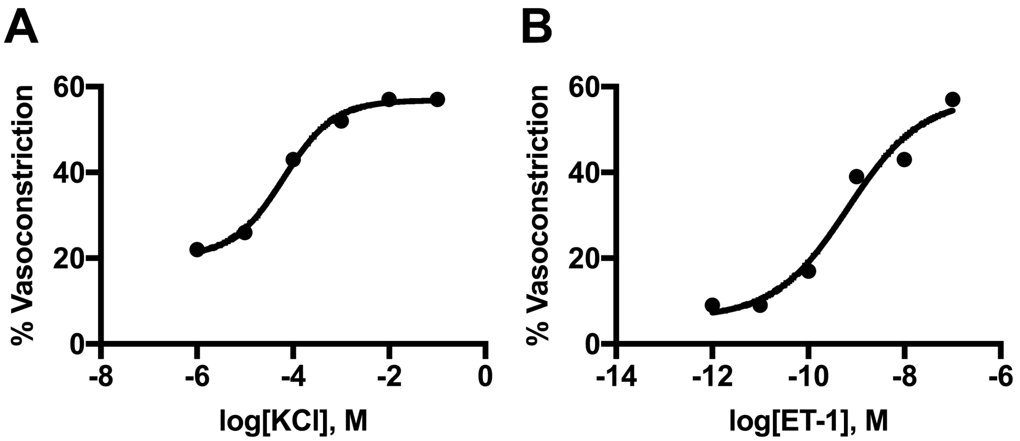

Supplementary Figure 1. Representative concentration-response curves for KCl and ET-1 obtained in the rat mesentery culture model. Representative concentration-response curves for KCl (A) and ET-1 (B) from Day 0 (pre-culture) tissues.

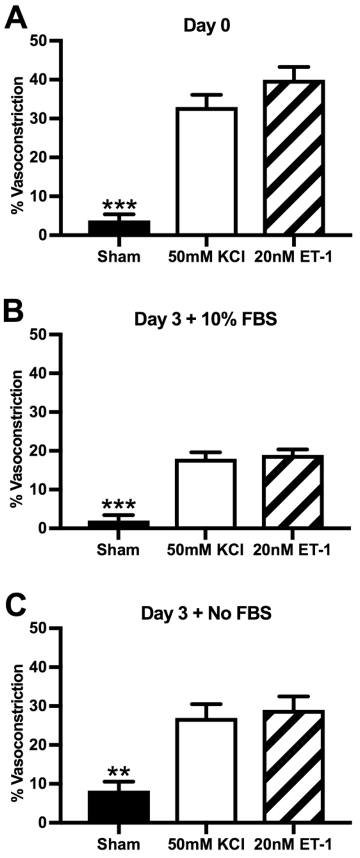

Supplementary Figure 2. Evaluation of arteriole vasoconstriction responses to sham controls for Day 0 (pre-culture) and Day 3 (cultured) tissues. A–C) Comparison of arteriole vasoconstriction from sham controls to 50 mM KCl and 20 nM ET-1, respectively. Arterioles from Day 0 (A) and Day 3 (B, C) groups exhibited a minimal vasoconstriction response to sham controls compared to 50 mM KCl and 20 nM ET-1 vasoconstrictors. Black, white, and striped bars represent Sham, 50 mM KCl, and 20 nM ET-1 groups respectively. ** and *** indicates a significant difference of $p < 0.01$ and $p < 0.001$ by One-Way ANOVA and Student-Newman-Keuls post hoc method.

Supplementary Video 1. Time-lapse video of 20 nM ET-1 vasoconstriction. Day 0 arteriole and venule constriction response to 20 nM ET-1 over a 5-minute period. Microvascular networks were visualized with BSI-lectin labeling. The original video frame rate (2 fps) has been increased to 30 fps. Arrows identify constriction along the length of the arteriole. A = arteriole, V = venule. Video was captured with a 10X (dry, NA = 0.3) objective.

Supplementary Video 2. Time-lapse video of 50 mM KCl vasoconstriction. Day 0 arteriole and venule constriction response to 50 mM KCl over a 5-minute period. Microvascular networks were visualized with BSI-lectin labeling. The original video frame rate (2 fps) has been increased to 30 fps. Arrows identify constriction along the length of the arteriole. A = arteriole, V = venule. Video was captured with a 10X (dry, NA = 0.3) objective.
